# lionessR: single sample network inference in R

**DOI:** 10.1186/s12885-019-6235-7

**Published:** 2019-10-25

**Authors:** Marieke L Kuijjer, Ping-Han Hsieh, John Quackenbush, Kimberly Glass

**Affiliations:** 1 Centre for Molecular Medicine Norway (NCMM), Nordic EMBL Partnership, University of Oslo, Gaustadalléen 21, Oslo, 0318 Norway; 2 Department of Biostatistics, Harvard T.H. Chan School of Public Health, 677 Huntington Ave, Boston, 02215 USA; 3 Channing Division of Network Medicine, Brigham and Women’s Hospital, 181 Longwood Avenue, Boston, 02215 USA; 4 Department of Medicine, Harvard Medical School, 25 Shattuck Street, Boston, 02215 USA

**Keywords:** Algorithms, Software tools, Computational biology, Biological networks, Network analysis, Co-expression, Gene regulation, Precision medicine, Osteosarcoma

## Abstract

**Background:**

In biomedical research, network inference algorithms are typically used to infer complex association patterns between biological entities, such as between genes or proteins, using data from a population. This resulting aggregate network, in essence, averages over the networks of those individuals in the population. LIONESS (Linear Interpolation to Obtain Network Estimates for Single Samples) is a method that can be used together with a network inference algorithm to extract networks for individual samples in a population. The method’s key characteristic is that, by modeling networks for individual samples in a data set, it can capture network heterogeneity in a population. LIONESS was originally made available as a function within the PANDA (Passing Attributes between Networks for Data Assimilation) regulatory network reconstruction framework. However, the LIONESS algorithm is generalizable and can be used to model single sample networks based on a wide range of network inference algorithms.

**Results:**

In this software article, we describe *lionessR*, an R implementation of LIONESS that can be applied to any network inference method in R that outputs a complete, weighted adjacency matrix. As an example, we provide a vignette of an application of *lionessR* to model single sample networks based on correlated gene expression in a bone cancer dataset. We show how the tool can be used to identify differential patterns of correlation between two groups of patients.

**Conclusions:**

We developed *lionessR*, an open source R package to model single sample networks. We show how *lionessR* can be used to inform us on potential precision medicine applications in cancer. The *lionessR* package is a user-friendly tool to perform such analyses. The package, which includes a vignette describing the application, is freely available at: https://github.com/kuijjerlab/lionessR and at: http://bioconductor.org/packages/lionessR.

## Background

Modeling and analyzing biological networks has become an invaluable tool in the analysis of genomic data. While gene expression profiles give us a snapshot of the state of a cell or tissue, network inference algorithms give an estimate of the extent to which genes or gene products interact [1]. Many network inference methods exist [2], most of which require multiple samples and population-level data to infer an “aggregate” condition-specific network [3–8]. These methods first construct a supervised model which then can be applied to single sample data [9–11]; however, they do not directly model networks for individual samples in a population.

We recently developed LIONESS, or Linear Interpolation to Obtain Network Estimates for Single Samples [12], as a way of using population-level networks to estimate the corresponding network in each individual sample. LIONESS is based on the idea that each sample has its own network and that each edge in an aggregate network is the “average” (a linear combination) of that edge’s weight across these individual sample networks. LIONESS starts by modeling an aggregate network on an entire population and then removes one sample and rebuilds the network. This is similar to leave-one-out cross-validation approaches [13]. However, LIONESS then compares the network with and without an individual sample, and uses a linear equation to estimate the network for the withheld sample. Thus, by sequentially leaving out each sample in a population, one can use LIONESS to estimate a network specific to each sample.

The LIONESS equation can be written as:

1 $$ e_{ij}^{(q)} = N\left(e_{ij}^{(\alpha)} - e_{ij}^{(\alpha-q)}\right) + e_{ij}^{(\alpha-q)}  $$

where $e_{ij}^{(\alpha)}$ is the weight of an edge between nodes *i* and *j* in a network modeled on all (*N*) samples and $e_{ij}^{(\alpha -q)}$ is the weight of that edge in a network modeled on all samples except the sample of interest (*q*).

Specifically, LIONESS subtracts edge weights, $e_{ij}^{(\alpha -q)}$, which are derived from a network modeled on all samples except the sample of interest (*q*), from edge weights, $e_{ij}^{(\alpha)}$, obtained from the network modeled on all samples; these differences represent the contribution of sample *q* to the aggregate network. With increasing numbers of samples in the aggregate network model, these contributions become smaller. LIONESS therefore scales these edge weight differences by multiplying them by *N*, the number of samples that were used to model the aggregate network. Finally, to estimate the single sample edge weights, $e_{ij}^{(q)}$, LIONESS adds the scaled edge weight differences, $N\left (e_{ij}^{(\alpha)} - e_{ij}^{(\alpha -q)}\right)$, to the edge weights obtained from the network modeled without the sample of interest, $e_{ij}^{(\alpha -q)}$. For more details on how we derived the LIONESS equation, please see the Supplemental Information section published in Kuijjer et al. [12].

LIONESS network estimation is included as an option to use with the PANDA network inference algorithm [7] in our Python tool PyPanda [14]. However, the LIONESS approach is not limited to modeling single sample PANDA networks—it can be used to model single sample networks based on a wide range of network inference algorithms. We developed *lionessR*, a user-friendly R implementation of LIONESS. The *lionessR* package can be used to estimate single sample networks for general network methods used in network and cancer biology, including Pearson correlation.

## Implementation

We developed the *lionessR* package in R using CRAN packages *devtools* and *roxygen2*. The package depends on R version >=3.0.2 and imports the CRAN library *stats*. The package is available as open-source code at https://github.com/kuijjerlab/lionessR and can be installed with *devtools*. Instructions for installation are given on the package’s GitHub site. In addition, an R package is available on Bioconductor at http://bioconductor.org/packages/lionessR.

Within the *lionessR* package, the lioness() function applies LIONESS (Eq. 1) to the output of a network inference algorithm, as defined by the function netFun(). The default network inference algorithm in netFun() is Pearson correlation, which builds correlation networks by returning an adjacency matrix of Pearson correlation coefficients. We included Pearson correlation as the default function, as correlation has been and continues to be widely used in many network applications [1, 2, 6, 7] and because correlation networks can be modeled on a wide variety of data types. However, netFun() can be substituted with any other uni- or bipartite network inference algorithm that returns a complete, weighted adjacency matrix. The lioness() function returns an R data frame that includes weights for all edges in each of the sample-specific networks.

The computation time of *lionessR* depends on the network reconstruction algorithm used in netFun(). *lionessR* calculates one aggregate network model based on all *N* samples, as well as *N* aggregate network models based on all samples except the sample of interest; therefore its computation time is *O*(*N*) times the computation time of modeling a “standard” aggregate network modeled with netFun(). For example, when using the default function netFun() (Pearson correlation) in *lionessR* on expression data of *M* genes, it takes *O*(*N*·*M*^2^) to compute all sample specific networks (if we assume arithmetic operations run in constant time).

The package comes with a vignette that shows how to model networks with *lionessR* and gives an example of how to analyze single sample *lionessR* networks. The vignette depends on the CRAN packages *igraph* and *reshape2* and the Bioconductor package *limma*. The package also includes an example dataset in the object OSdata, which includes expression data for pre-operative osteosarcoma biopsies from 53 high-grade osteosarcoma patients, as well as information on whether patients developed metastases within five years since diagnosis of the primary tumor. These data were obtained from the Gene Expression Omnibus (GEO, accession GSE42352), and included samples with at least 70% tumor content and viability, for which RNA was profiled on Illumina human-6 v2.0 microarray beadchips and pre-processed using Bioconductor package *lumi* [15], as previously described [16]. The example data are used in the vignette to model single sample networks for the 53 patients based on correlation networks. The workflow of modeling these individual patient networks and of analyzing them in the context of metastasis-free survival is given in the Results section below.

## Results

### Application of *lionessR* to a bone cancer dataset

As an example, we performed an analysis applying lioness() to a gene expression dataset from 53 high-grade osteosarcoma biopsies [16] (Gene Expression Omnibus accession number GSE42352), which is included with the package. High-grade osteosarcoma is an aggressive primary bone tumor that has peak incidence in adolescents and young adults. About 45% of patients develop metastases, and most metastatic patients eventually die from the disease [17]. We performed a differential correlation network analysis comparing short- versus long-term metastasis-free survival (MFS) to understand co-regulation differences between the groups and to search for potential therapeutic targets.

For this demonstration, we separated patients into two groups based on those who developed metastases within five years (*n*=19) and those who did not (*n*=34). These were the same groups analyzed by Buddingh et al. [18] to compare gene expression levels between short- and long-term MFS. To decrease the runtime of our tutorial application, we limited our analysis to the 500 most variable genes based on the standard deviation. We used lioness() to model 53 single sample networks based on Pearson correlation, one for each individual in the population, using the entire population to estimate the background network, with the code:cormat <- lioness(dat, netFun),where dat is the input expression data and cormat the lioness output.

### Comparative analysis of single sample bone cancer networks modeled with *lionessR*

**Fig. 1:**
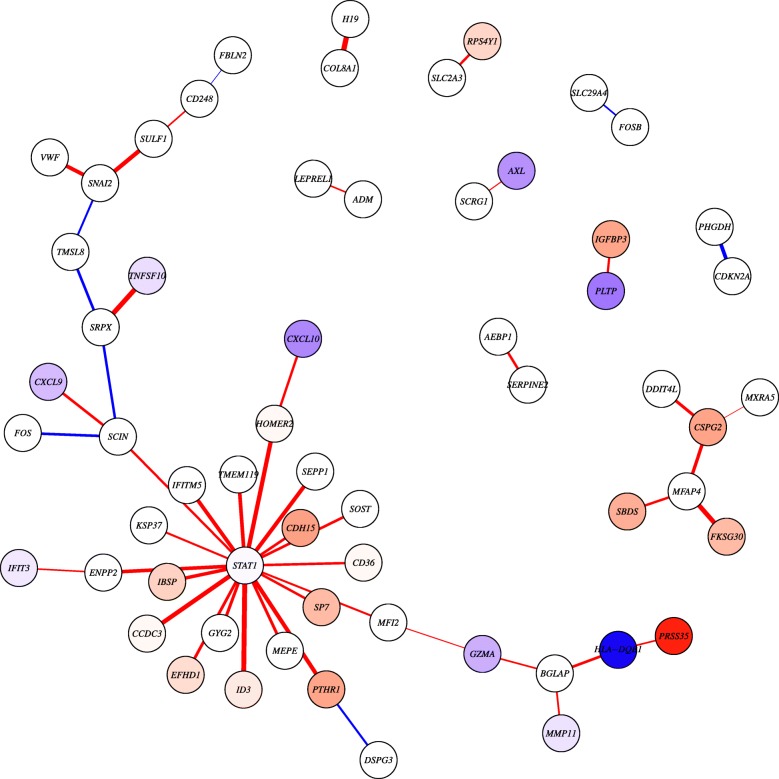
Significant differential network edges associated with osteosarcoma survival. Network visualization of the 50 edges with the most significant differences in their estimated correlation based on a LIMMA analysis comparing single sample edge weights between patients with poor and better MFS. Edges are colored based on whether they have higher weights in patients with poor (red) or better (blue) MFS. Thicker edges represent higher fold changes. Absolute edge fold changes range from [0.75,1.28]. Nodes (genes) are colored based on the t-statistic from a differential expression analysis. Nodes with absolute t-statistic <1.5 are shown in white, nodes in red/blue have higher expression in patients with poor/better MFS, respectively

We asked whether there were differences in network edge weights between the short- and long-term MFS groups. As the aggregate network model in this demonstration is Pearson correlation, a large edge weight in a single sample network indicates that adding that sample increases the Pearson correlation of the aggregate network, while a low edge weight means that addition of the sample decreases the aggregate network’s correlation coefficient for that edge. To reduce the number of statistical tests on these networks ($500 \choose 2=124750$ potential edges), we modeled two condition-specific networks and selected those edges that had an edge weight difference of at least 0.5 between these two networks. We then performed a LIMMA analysis [19] to identify those edges whose weights differed significantly between the groups. In parallel, we also used LIMMA to test for significant differences in gene expression levels between groups. We visualized the 50 most significantly perturbed edges (all nominal *p*<0.001, *FDR*<0.15) in a network diagram (Fig. 1).

We identified multiple significant differential connections to genes encoding for extracellular matrix proteins, including *BGLAP*. *BGLAP* encodes for osteocalcin, a protein secreted by osteoblasts to regulate bone remodeling. *BGLAP* was connected to both a matrix metalloproteinase (*MMP11*), involved in breakdown of extracellular matrix, and to genes involved in the immune system—*GZMA* and *HLA-DQB1*. *GZMA* encodes for Granzyme A, a T-cell and natural killer cell-specific protease, while *HLA-DQB1* is a Matrix Histocompatibility Complex (MHC) Class II gene involved in antigen presentation. All of the edges connected to *BGLAP* had a moderate to strong negative correlation (range *R*=[−0.75,−0.61]) in the samples with better MFS, whereas these edges had a weak positive correlation (range *R*=[0.22,0.26]) in the poor MFS group.

Interestingly, *BGLAP* was not differentially expressed between these groups (log fold change (logFC) =0.23, *p*=0.68). This indicates that these processes are tightly regulated in tumors of patients with long-term survival and that loss of this regulation is associated with worse outcome. It also suggests a link between matrix remodeling and recruitment of immune cells, which could indicate that bone remodeling in osteosarcoma may result in the recruitment of immune cells to clear up the cancer, confirming previous findings of osteoclast [20] and macrophage [18] association with MFS in osteosarcoma.

In addition, we identified a highly connected gene, or network “hub,” among the nodes connected to the top 50 edges—*STAT1*, or Signal Transducer And Activator Of Transcription 1. *STAT1* is a transcription factor and thus potentially differentially regulates the target genes with which it is correlated. In fact, all of the edges connected to *STAT1* had a moderate to strong negative correlation (range *R*=[−0.84,−0.42], median *R*=−0.67) in the samples with better MFS, whereas these edges had a weak to moderate positive correlation (range *R*=[0.14,0.42], median *R*=0.30) in the poor MFS group. This suggests that *STAT1* may repress expression of these genes in patients with long-term MFS. However, this repression is lost in patients with short-term MFS. It has been previously shown that in tumors with good prognosis, high *STAT1* expression inhibits bone formation [21]. The target genes we identified that connect to *STAT1* (Figure 1) were enriched for being annotated to the Gene Ontology term “ossification,” (Fisher’s exact test odds ratio=5.76, p-value=0.0056), which is consistent with this result. These genes included *SOST*, *SP7*, *IBSP*, *IFITM5*, and *TMEM119*.

More importantly, *STAT1* is a transcription factor in the interferon signaling pathway—a pathway known to be involved in osteosarcoma, and for which targeted treatment options are available [22]. This indicates that individual patient correlation network analysis with *lionessR* can pinpoint potential candidates for personalized medicine. Importantly, *STAT1* is not differentially expressed itself (logFC =0.44, *p*=0.19) and neither are many of its target genes. Thus, we would not have been able to obtain this result by analyzing differential expression alone, without placing these genes into a framework of a network. In fact, we previously identified differential gene regulation in the absence of differential expression by analyzing LIONESS networks modeled based on the PANDA [7] network reconstruction framework, which suggested a potential mechanism for sexual dimorphism in colorectal patients [23]. The current example in osteosarcoma highlights the potential of *lionessR* in modeling networks for individual cancer patients based on other network inference approaches.

## Conclusions

Precision medicine uses data about the state of individual genes to match each patient to the therapies that are most likely to be efficacious for them. However, even when therapies target a specific gene mutation, we know that many patients who carry a particular mutation, or whose gene expression signatures correspond to known response biomarkers, do not always respond to targeted treatment. Clearly, to improve precision medicine, we need to better understand the complex relationships that exist between different genes and gene products in individual samples. Networks are a natural way to represent these complex interactions, but methods to infer networks generally “average” over the members of a population. Using networks in precision medicine requires methods that allow inference of network models specific to each individual, reflecting the heterogeneity in the population.

LIONESS represents a method that can fill the gap between methods that infer networks using population data and the need for methods that can model networks specific to each individual. LIONESS estimates individual sample networks by using linear interpolation iteratively, extracting a network for each member of a population [12]. LIONESS essentially measures how removing a single individual from a population changes the aggregate network, and uses those changes to identify the most likely network for that individual. The *lionessR* package allows users to apply this method in combination with different network inference algorithms, including Pearson correlation.

As an example, we modeled single sample networks based on the 500 genes with the highest variability in expression in an osteosarcoma dataset. We divided this dataset into two groups—patients with either short-term or long-term MFS. Comparing these two collections of networks using a LIMMA analysis, we identified *STAT1* to be significantly co-expressed with a set of “target” genes in biopsies of patients with poor survival. This set of genes was highly associated with biological processes important in osteosarcoma. In addition, *STAT1* is part of a biological pathway for which targeted treatment is available. This example highlights how single sample correlation network analysis can be used to inform us on potential precision medicine applications. The *lionessR* package is a user-friendly tool to perform such analyses.

## Availability and requirements

**Project name**: lionessR**Project home page**: https://github.com/kuijjerlab/lionessR**Operating system(s)**: Platform independent**Programming language**: R**Other requirements**: The vignette walkthrough requires the following R packages: devtools, igraph, reshape2, limma**License**: CC-BY-4.0**Any restrictions to use by non-academics**: None

## Data Availability

All data analyzed in this study are available in the *lionessR* package on GitHub: https://github.com/kuijjerlab/lionessR

## Abbreviations

LIONESS: Linear Interpolation to Obtain Network Estimates for Single Samples
MFS: Metastasis-free survival
PANDA: Passing Attributes between Networks for Data Assimilation
